# Supplementary material for: Deleterious mutation accumulation and the long-term fate of chromosomal inversions
Source: PLoS Genet. 2021 Mar 4;17(3):e1009411. doi: 10.1371/journal.pgen.1009411 (PMC7963061; doi:10.1371/journal.pgen.1009411)
Supplement: S1 Fig — A) the mutational load in the whole population at the end of the burn-in. B) the mutational load of the inverted arrangement in the haplotypes we selected (200 random plus the 4 best and the 4 worst and one close to the median). C) the mutational load of the inverted arrangement after correcting for the number of simulations done per haplotype. This figure illustrates that we do not always have the same number of simulations for each datapoint in Fig 1. (PDF) [file pgen.1009411.s001.pdf]

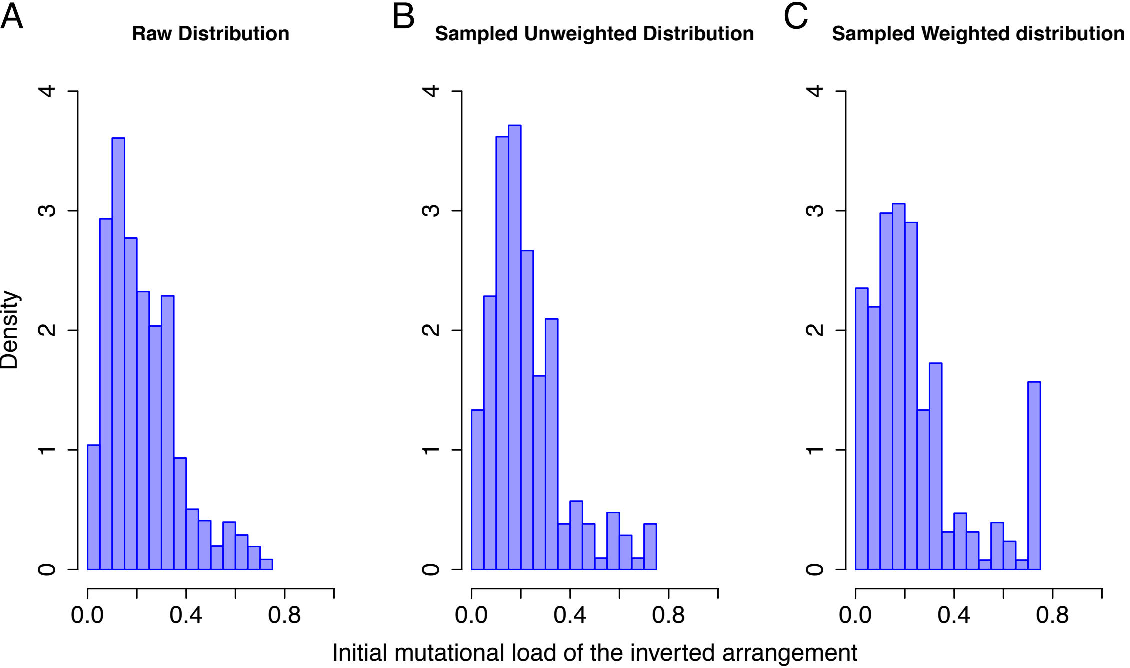
A
Raw Distribution
B
Sampled Unweighted Distribution
C
Sampled Weighted distribution
Density
0
1
2
3
4
0.0
0.4
0.8
Initial mutational load of the inverted arrangement
