# Supplementary figures and images for: Deleterious mutation accumulation and the long-term fate of chromosomal inversions
Source: PLoS Genet. 2021 Mar 4;17(3):e1009411. doi: 10.1371/journal.pgen.1009411 (PMC7963061; doi:10.1371/journal.pgen.1009411)

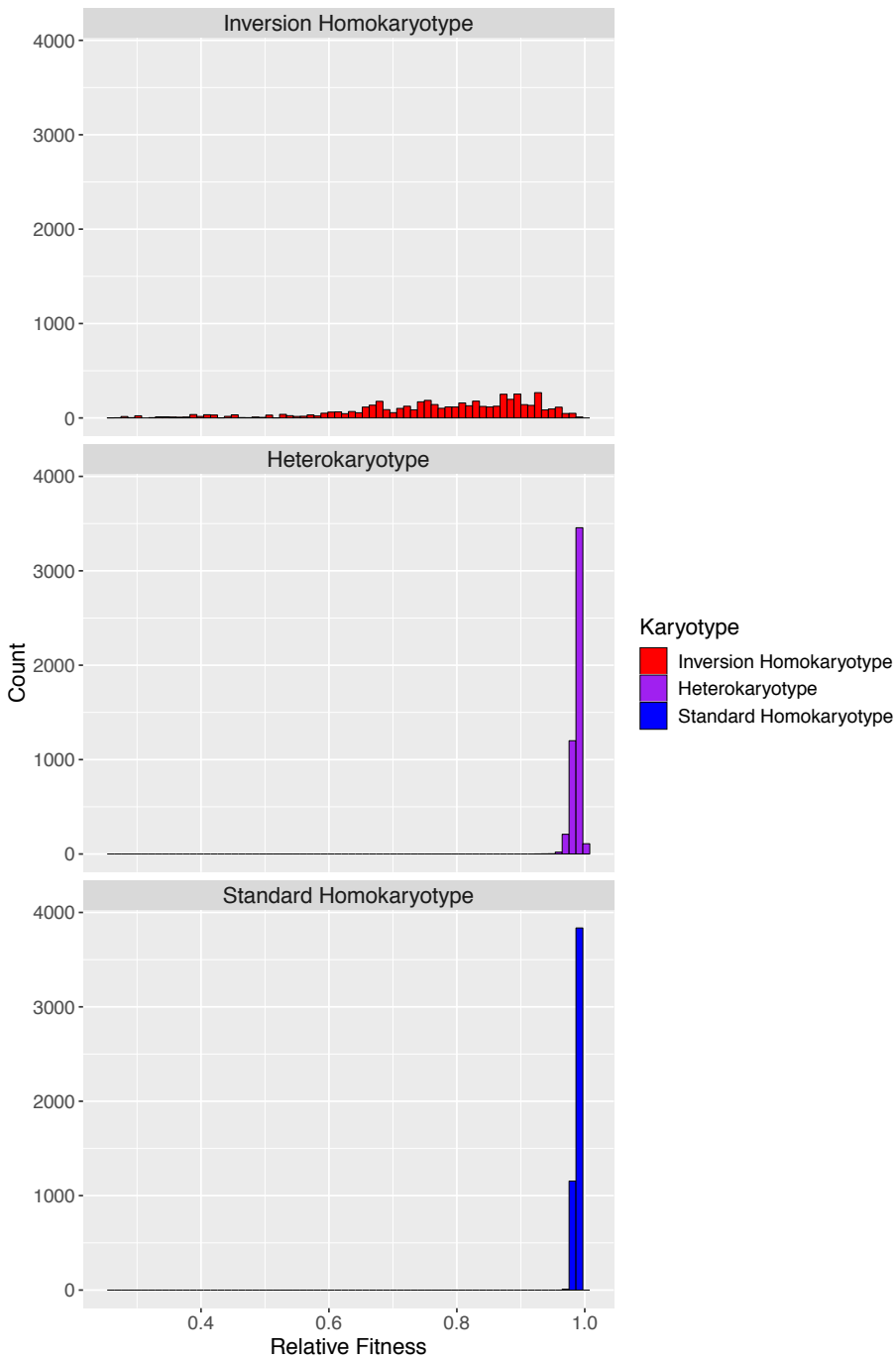

Supplement: S2 Fig — (PDF) [file pgen.1009411.s002.pdf]

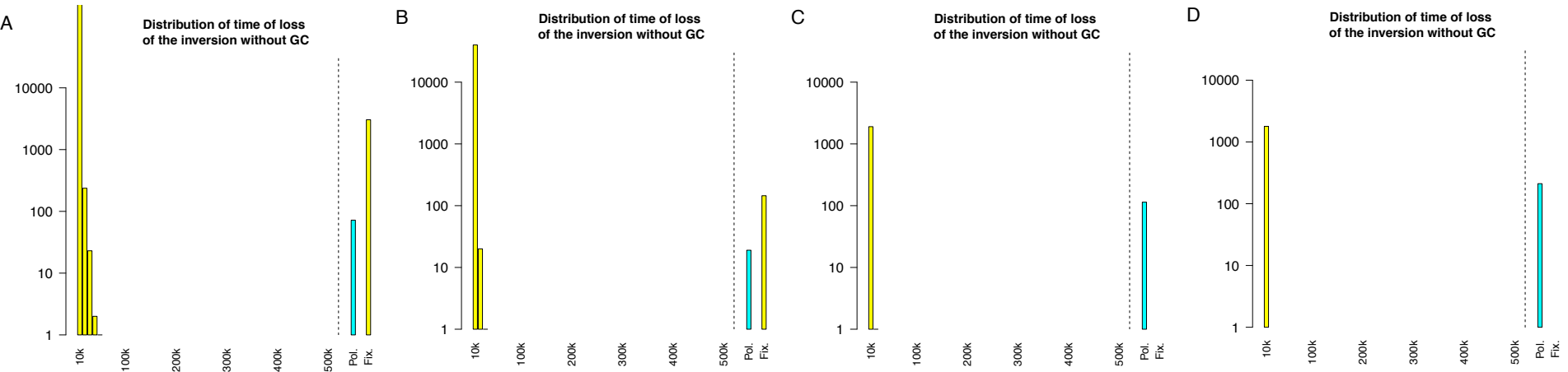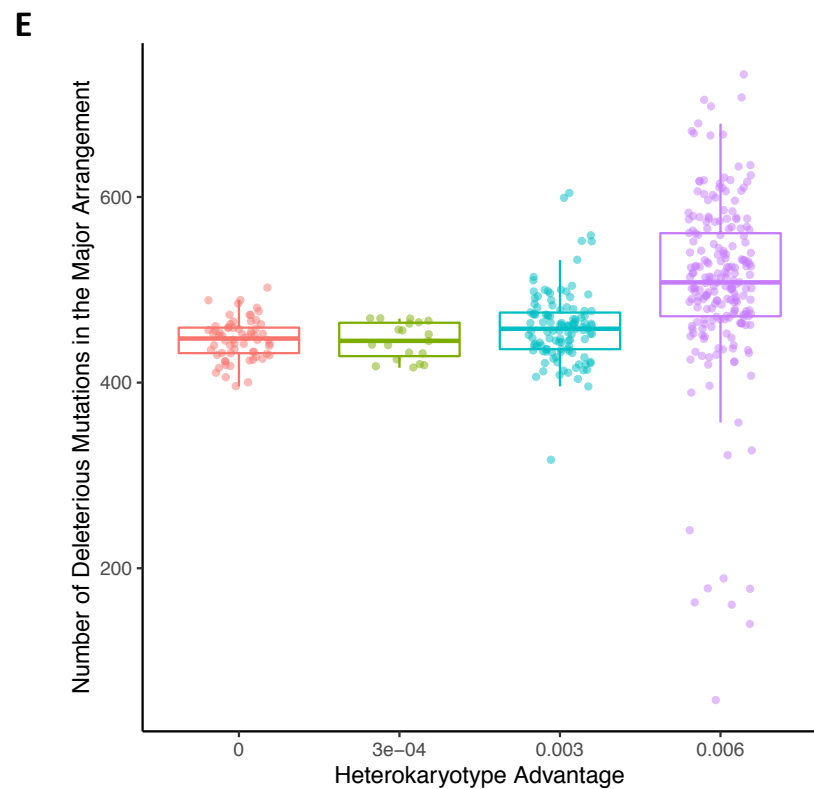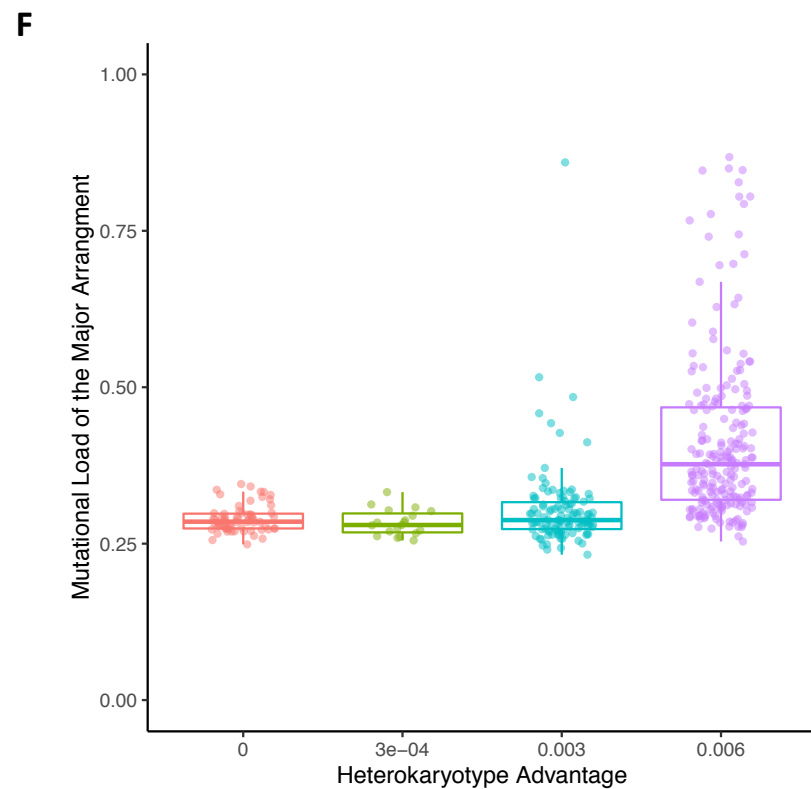

Supplement: S3 Fig — (A) Distribution of the time of loss of the inversion at sHET = 0. The number of simulations that remained polymorphic (cyan) or fixed (yellow) are indicated specifically to the right of the dashed line. (B) Distribution of the time of loss of the inversion at sHET = 0.0003. The number of simulations that remained polymorphic (cyan) or fixed (yellow) are indicated specifically to the right of the dashed line. (C) Distribution of the time of loss of the inversion at sHET = 0.003. The number of simulations that remained polymorphic (cyan) or fixed (yellow) are indicated specifically to the right of the dashed line. (D) Distribution of the time of loss of the inversion at sHET = 0.006. Simulations that remained polymorphic (cyan) or fixed (yellow) are indicated specifically to the right of the dashed line. (C) Mutation accumulation in the major arrangement under sHET = 0 (red), sHET = 0.0003 (green), sHET = 0.003 (cyan), and sHET = 0.006 (purple). Each dot represents a single run that ended at generation 500,000. (D) Mutation accumulation in the major arrangement under sHET = 0 (red), sHET = 0.0003 (green), sHET = 0.003 (cyan), and sHET = 0.006 (purple). Each dot represents a single run that ended at generation 500,000. (PDF) [file pgen.1009411.s003.pdf]

**A**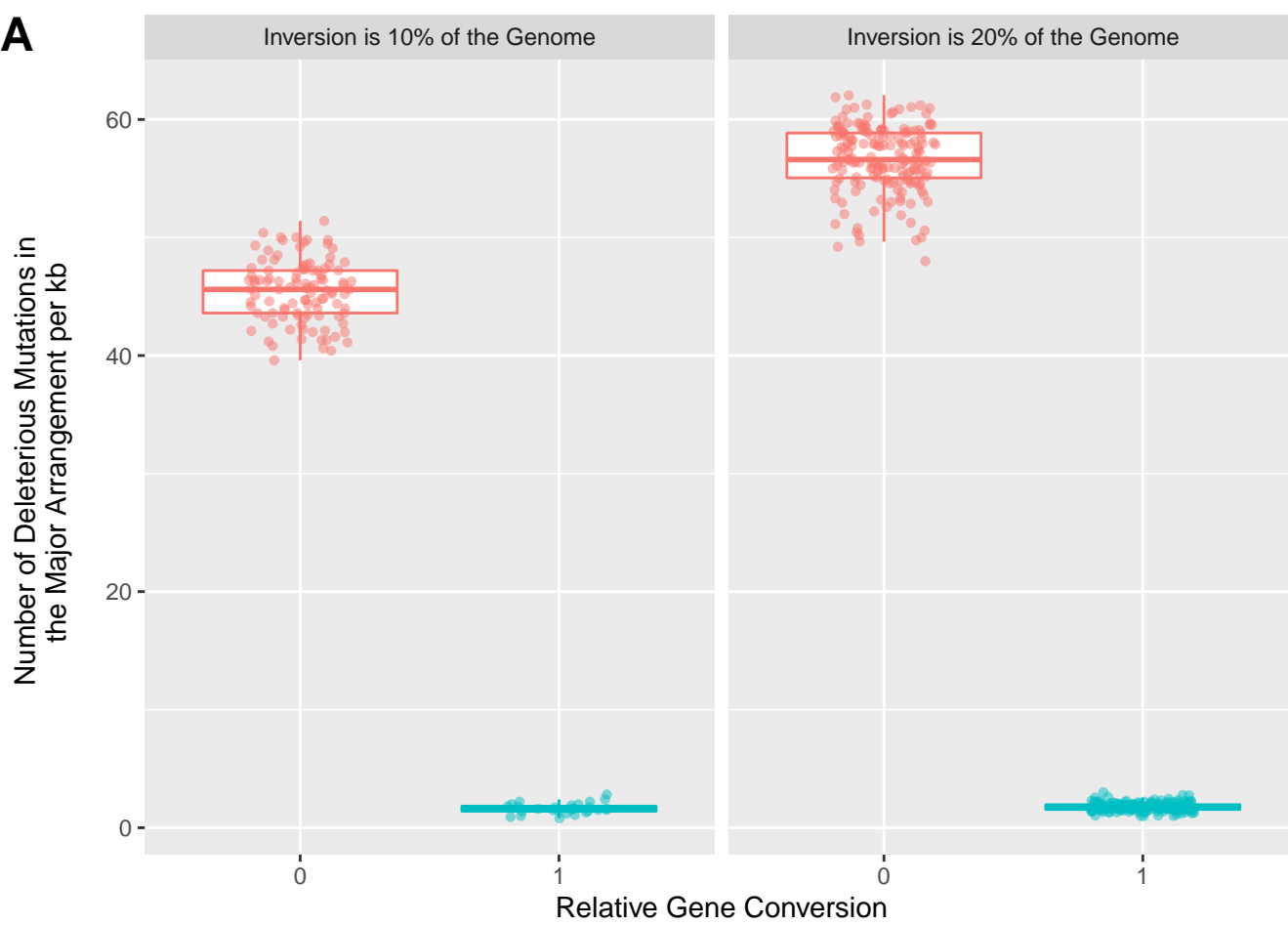**B**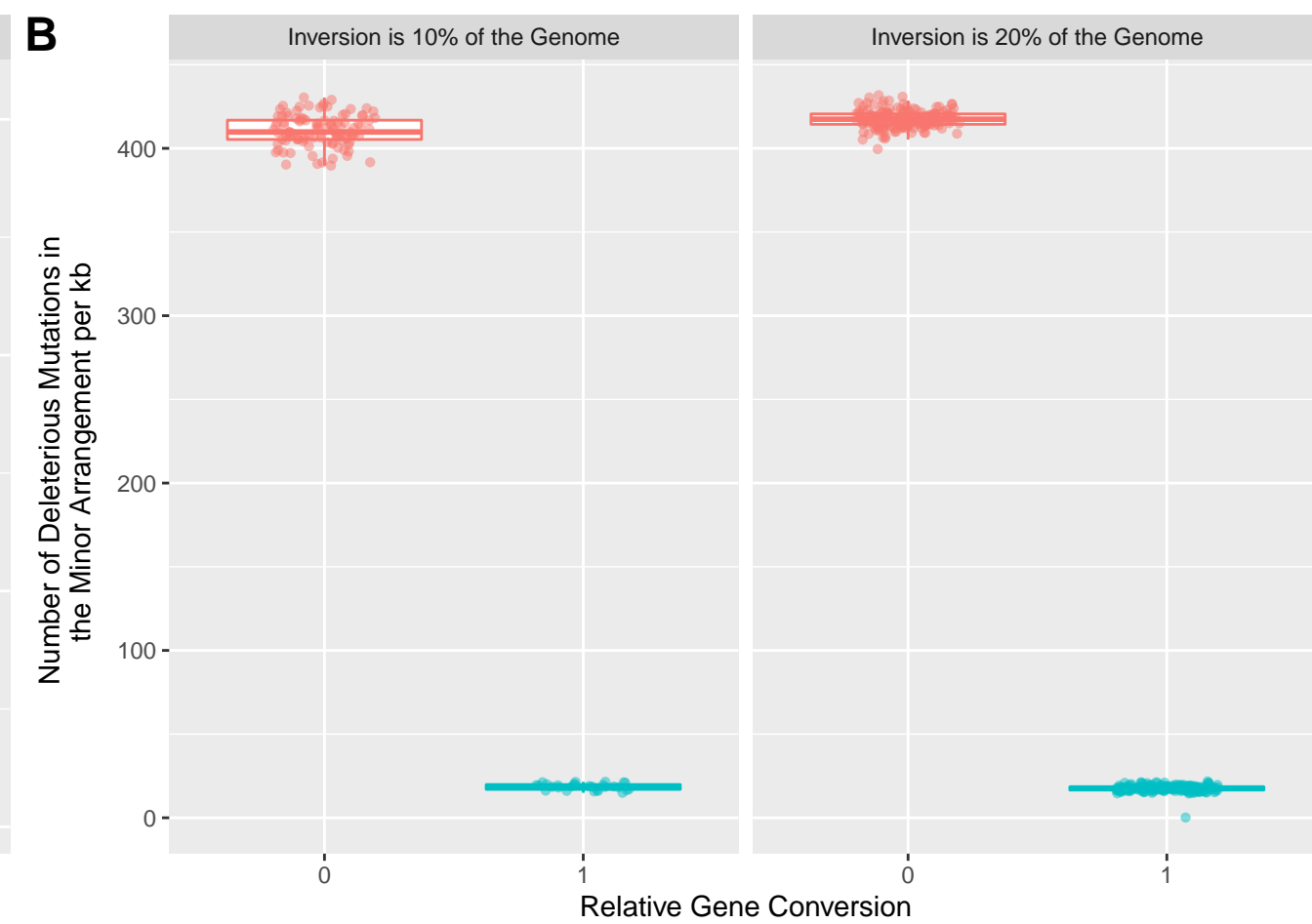**C**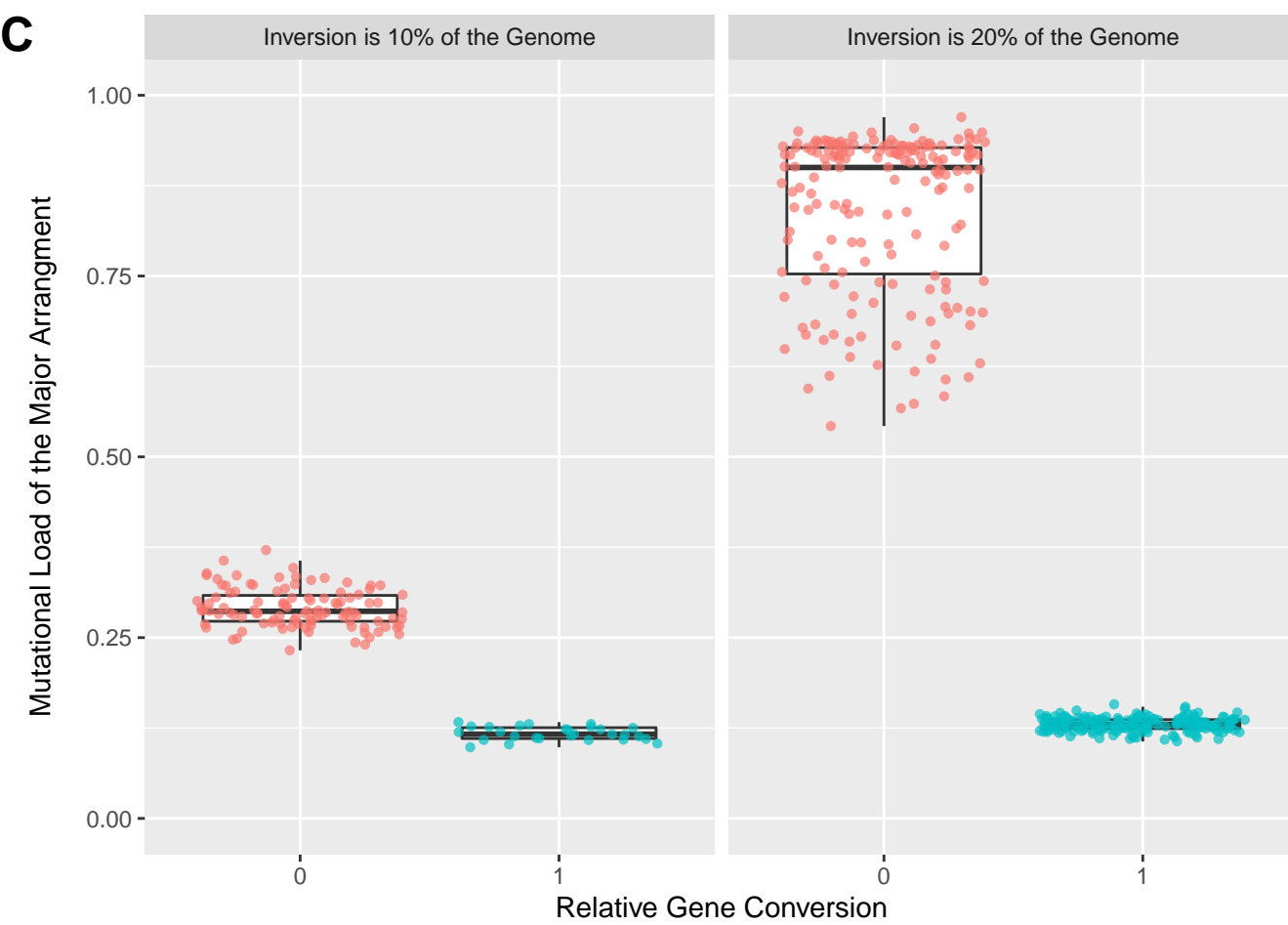**D**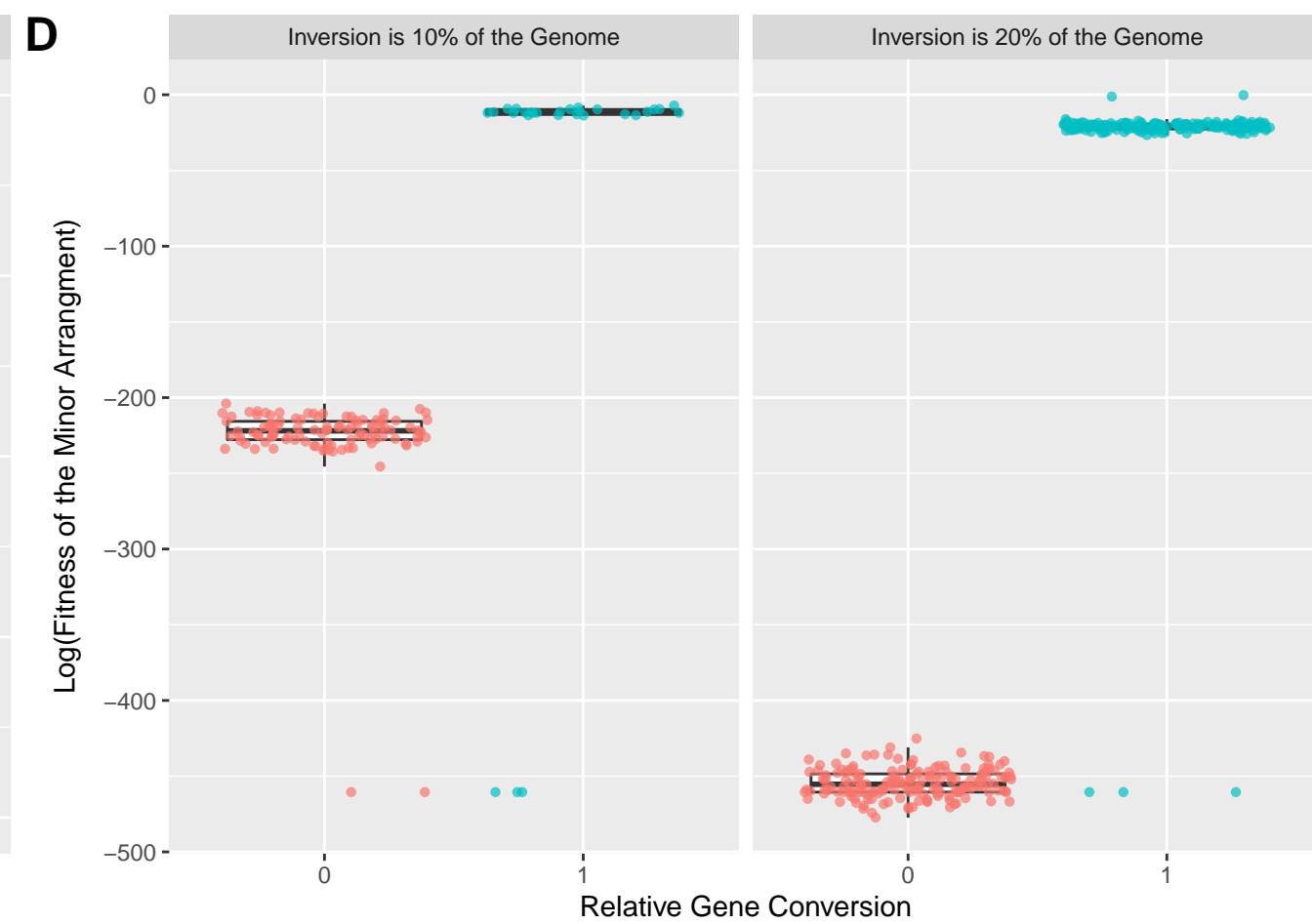

Supplement: S5 Fig — Mutation accumulation (A,B) and Mutational load (C,D) for the major (A,C) and minor (B,D) arrangements for different sized inversions. Color indicates presence (red) or absence (blue) of gene conversion. Each dot represents a single run. (PDF) [file pgen.1009411.s005.pdf]

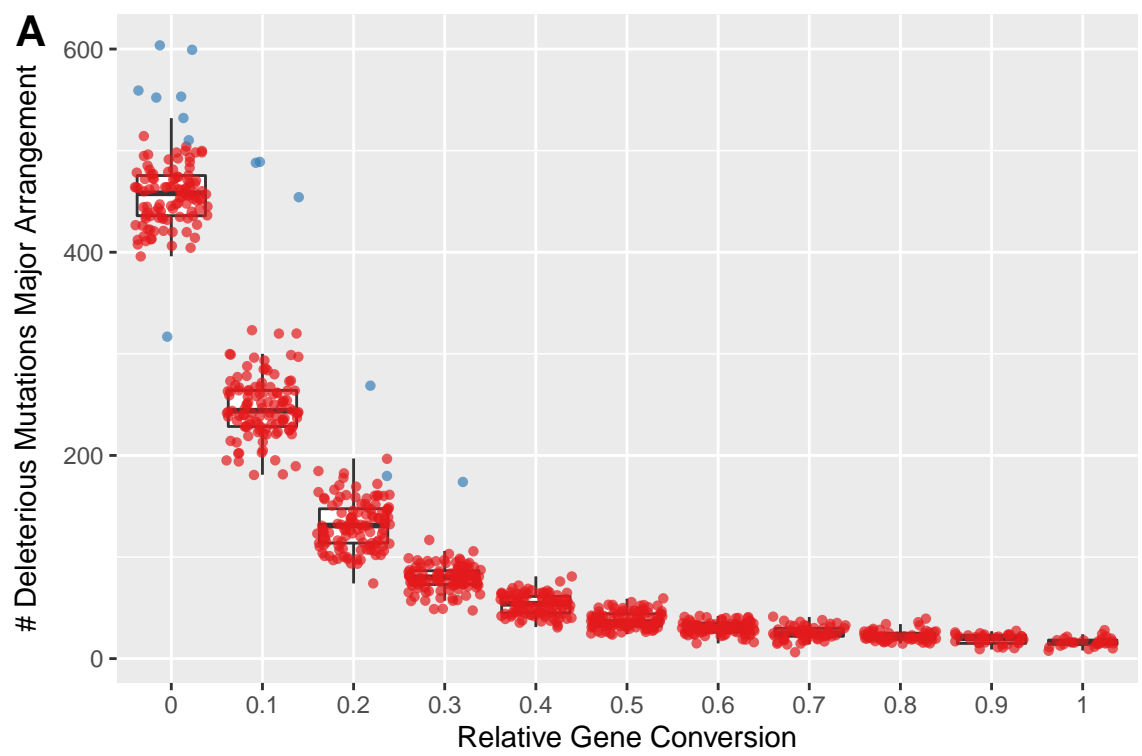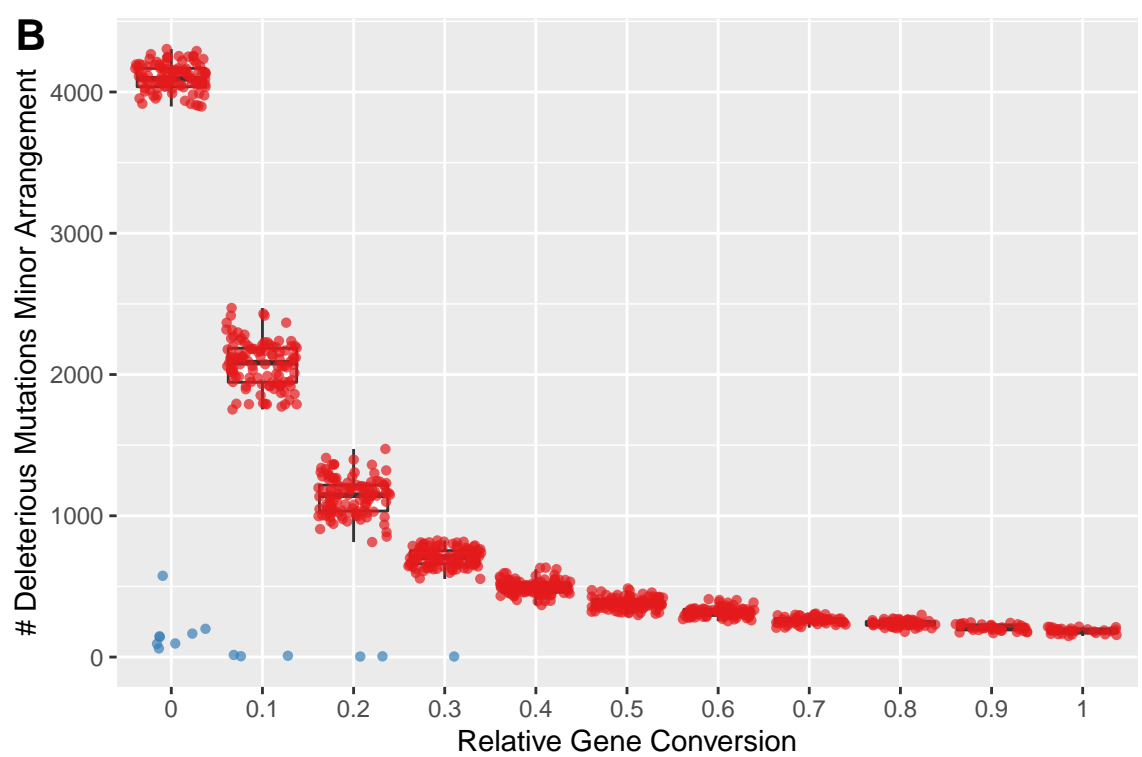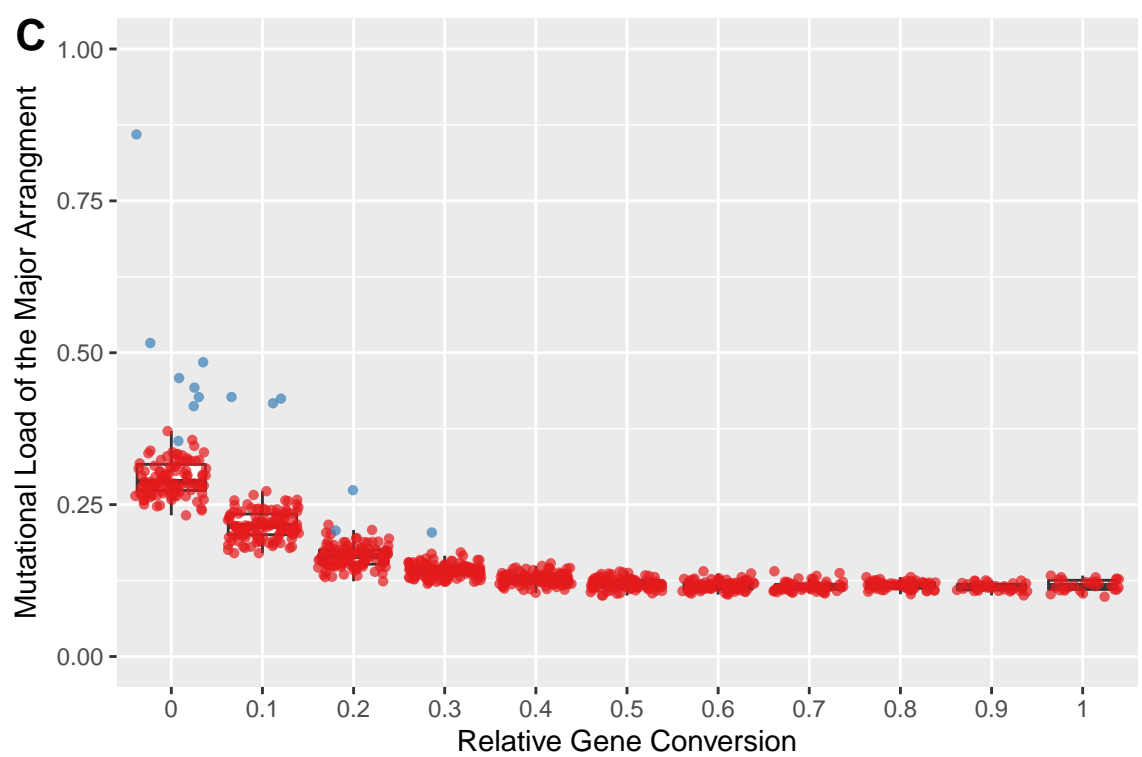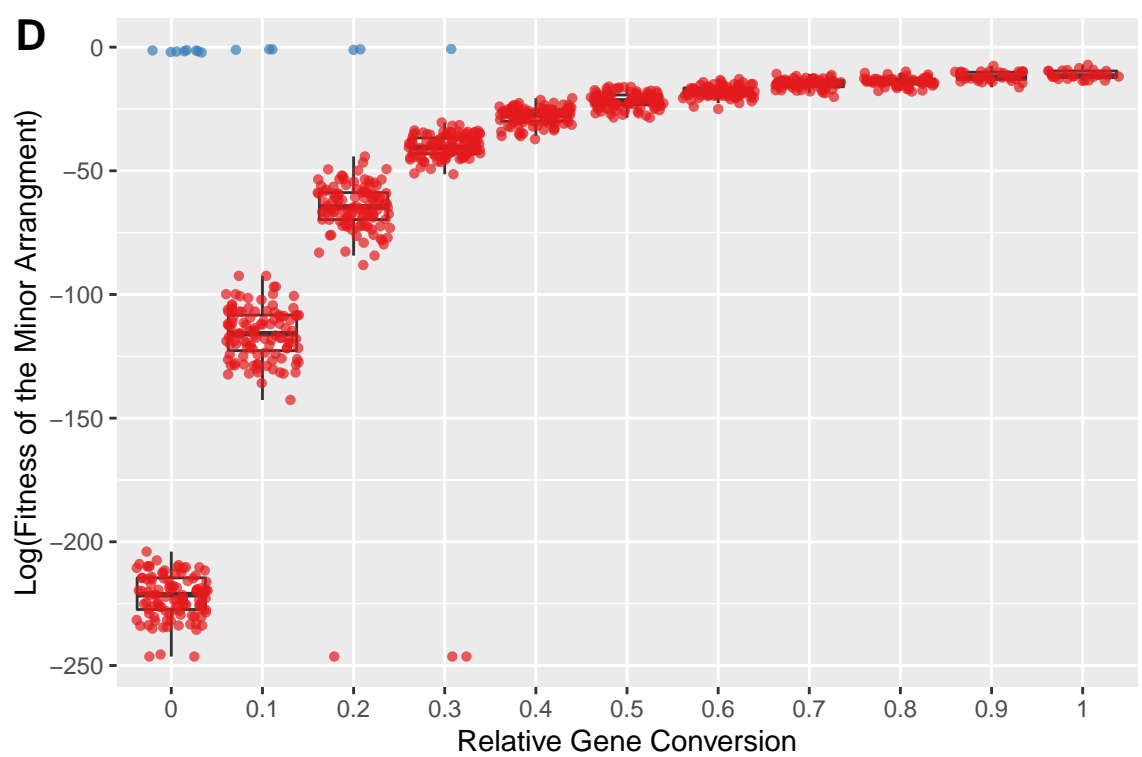

Supplement: S6 Fig — Gene Conversion exponentially affects mutation accumulation and mutational load of the arrangements. (A) Boxplot showing the number of deleterious mutations accumulated in the major arrangement after 500,000 generations. Overlain points represent single runs where haplotype structuring did not occur (red) or did occur (blue). (B) Boxplot showing the number of deleterious mutations accumulated in the minor arrangement after 500,000 generations. Overlain points represent single runs where haplotype structuring did not occur (red) or did occur (blue). (C) Boxplot showing the mutational load of the major arrangement after 500,000 generations. Overlain points represent single runs where haplotype structuring did not occur (red) or did occur (blue). (D) Boxplot showing the log fitness the minor arrangement after 500,000 generations. Overlain points represent single runs where haplotype structuring did not occur (red) or did occur (blue). Five points, which were zero due to R’s internal cutoff, were replaced by 1 x 10^−7^. (PDF) [file pgen.1009411.s006.pdf]

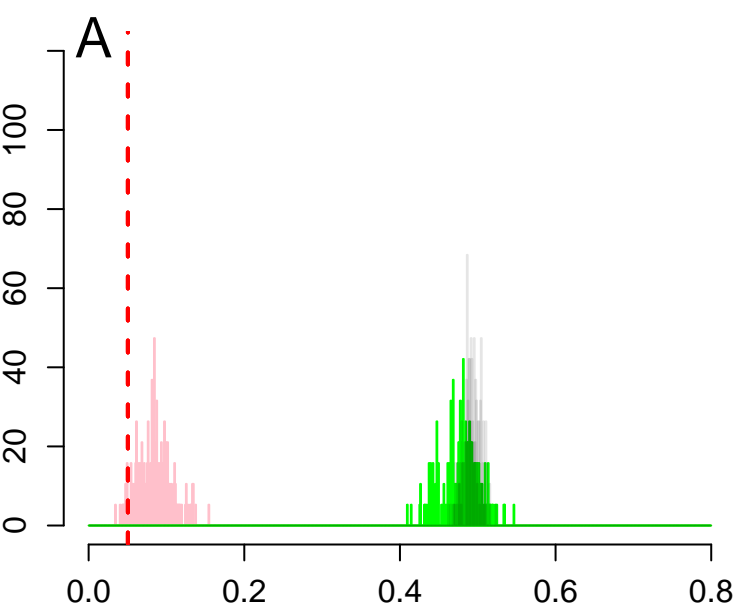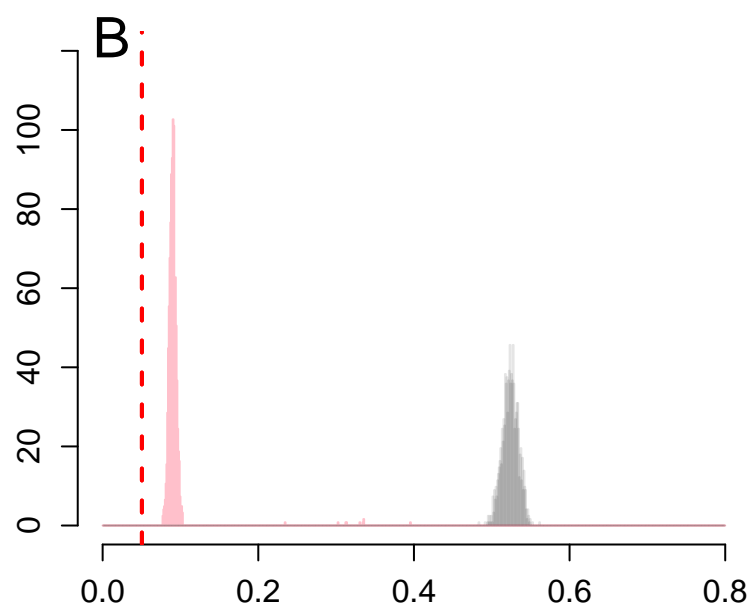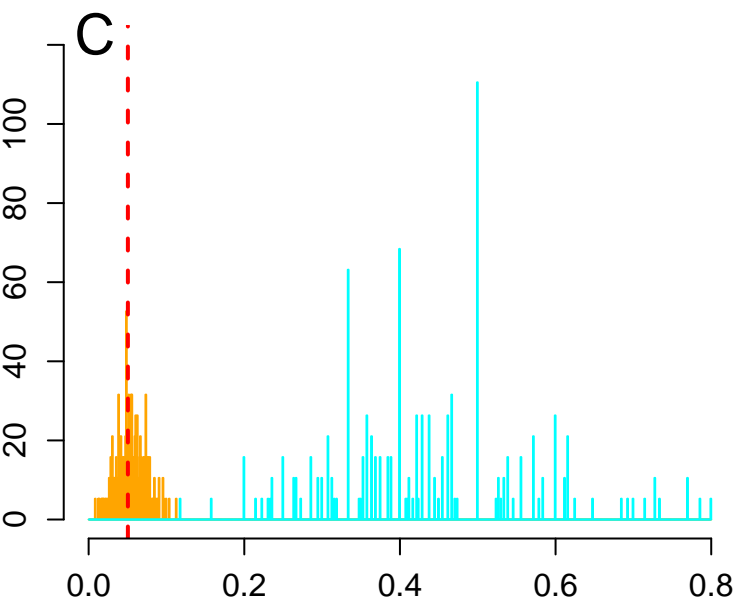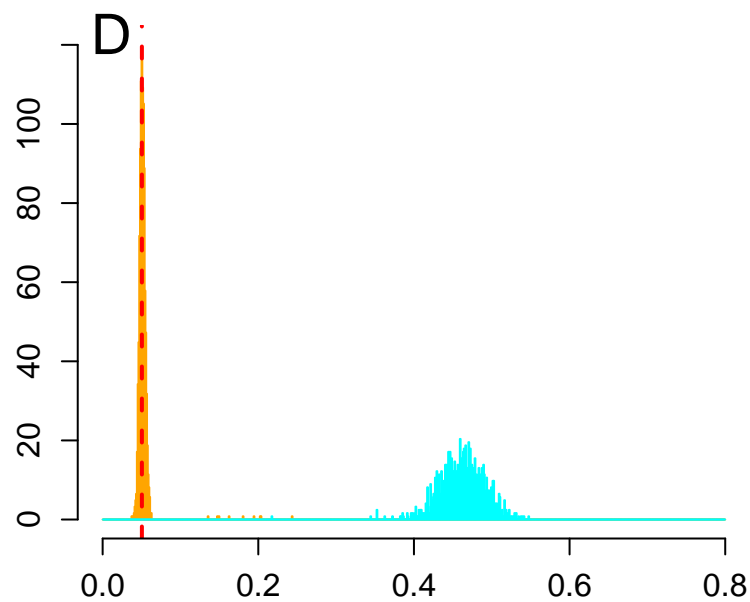

Proportion of effectively neutral alleles

Proportion of effectively neutral alleles

Supplement: S7 Fig — Distribution of proportion of effectively neutral alleles among fixed mutations with (B,D) and without (A,C) gene conversion. Orange corresponds to mutations fixed in minor arrangement, cyan to mutations fixed in the major arrangement, pink to the average of mutations fixed in either the major or minor arrangement (i.e. alleles with an FST of 1), green to mutations that have fixed in the inverted region (i.e. fixed in both arrangements), and black to mutations that have fixed in the collinear region (chromosomes 2 and 3). The dashed black line indicate the proportion of new mutations that are effectively neutral, and the red dashed line corresponds to the proportion of effectively neutral mutations that fixed during the burn-in. (PDF) [file pgen.1009411.s007.pdf]

A

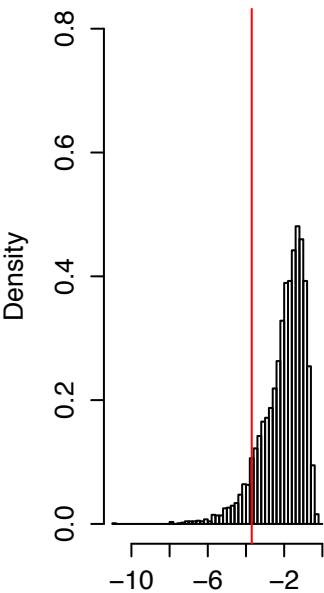

B

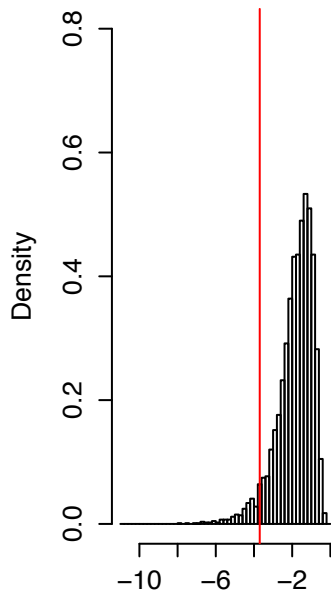

C

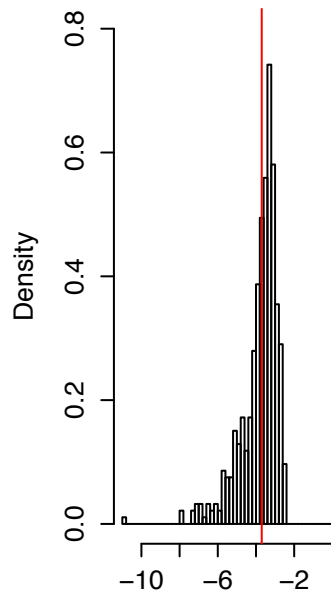

log(-selective coefficient)

Supplement: S8 Fig — The red line indicates s = 1/(2N); to the left mutation are effectively neutral. A) All deleterious mutations within the inverted region, B) all deleterious mutations private to and fixed in the minority arrangement and C) all deleterious mutations private to and fixed in the majority arrangement. (PDF) [file pgen.1009411.s008.pdf]

**A**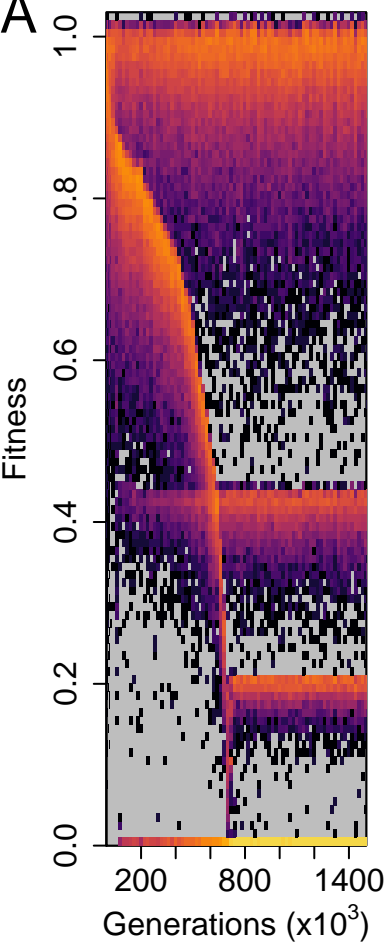**B**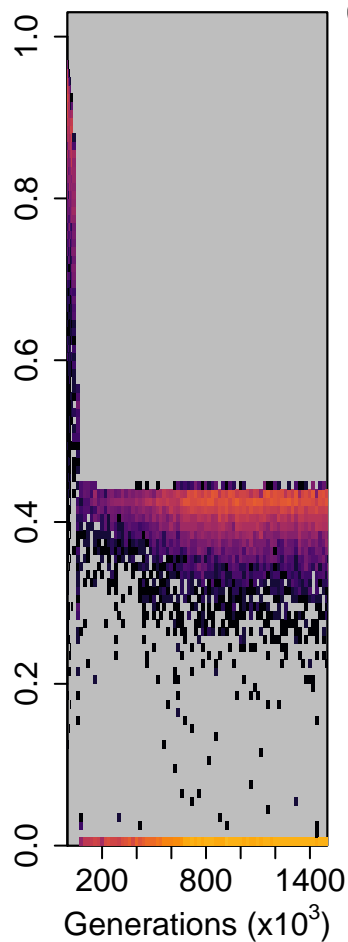**C**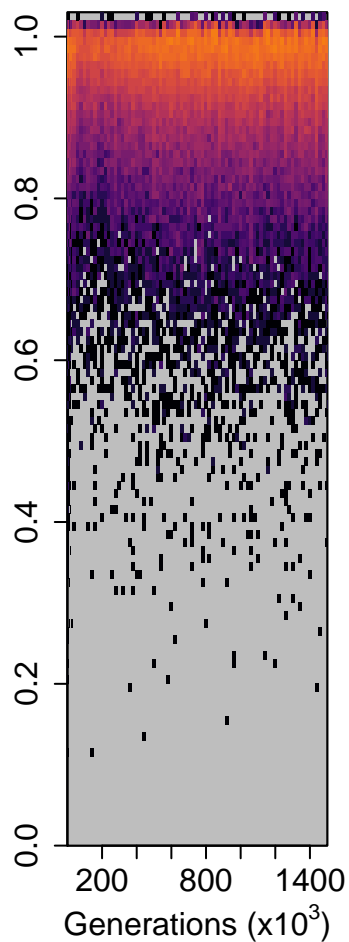**D**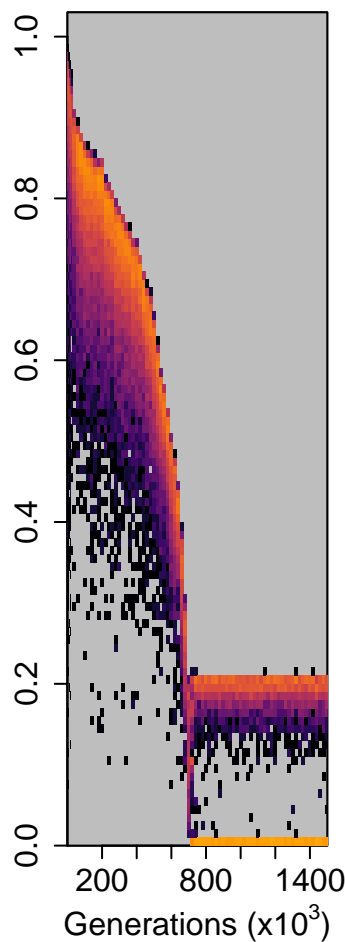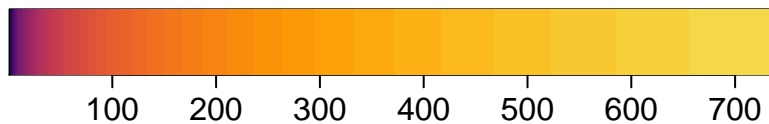

Number of individuals

Supplement: S9 Fig — The different panels correspond to the fitness distribution of A) the whole population, B) the inversion homokaryotype, C) the heterokaryotype and D) the standard homokaryotype. The color indicates how many individuals share a given fitness values (on a log scale). (PDF) [file pgen.1009411.s009.pdf]

**A**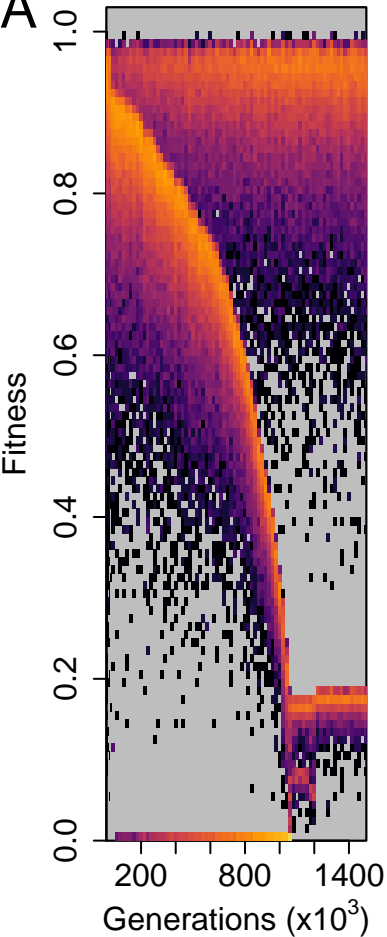**B**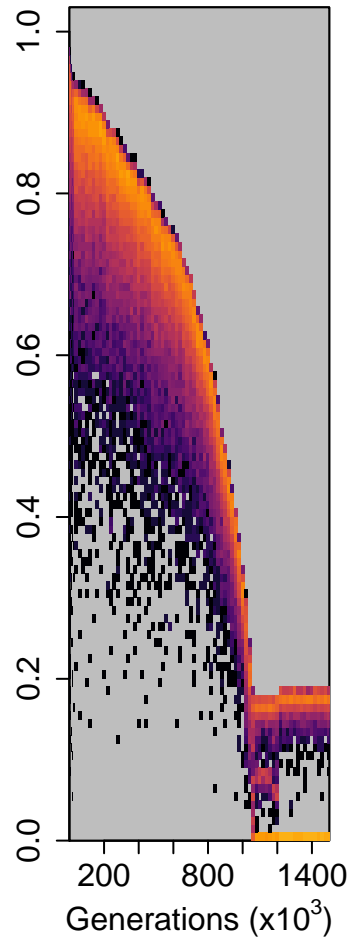**C**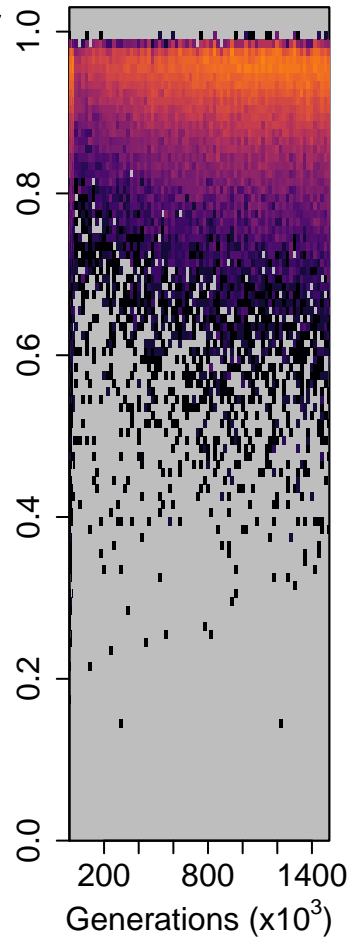**D**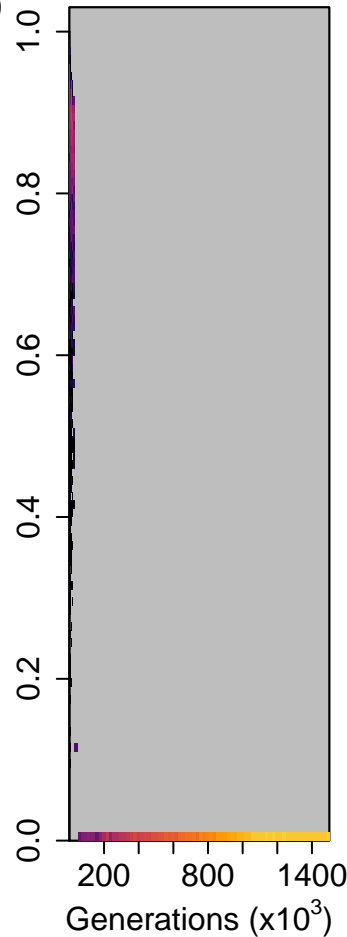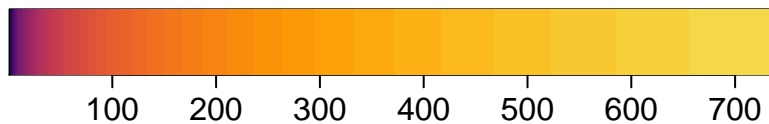

Number of individuals

Supplement: S10 Fig — The different panels correspond to the fitness distribution of A) the whole population, B) the inversion homokaryotype, C) the heterokaryotype and D) the standard homokaryotype. The color indicates how many individuals share a given fitness value (on a log scale). (PDF) [file pgen.1009411.s010.pdf]

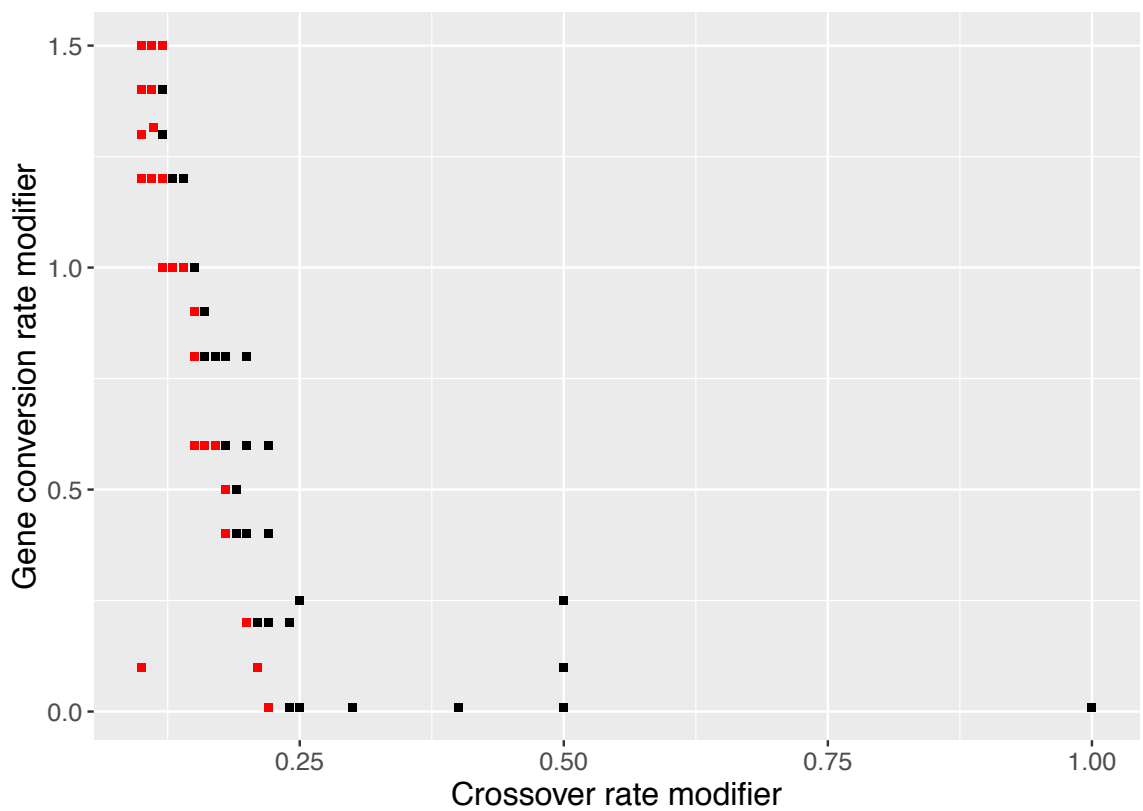

Supplement: S11 Fig — We consider a chromosome without an inversion but sharing the same properties than our inversion model (see Materials and Methods for details) and determine the combination of crossing over and gene conversion rate where we observe haplotype structuring in at least 1 of 10 replicates (in red; black indicates that haplotype structuring was not observed). The X and Y axis corresponds to the relative values of crossing over and gene conversion rate compared to the main simulations. (PDF) [file pgen.1009411.s011.pdf]

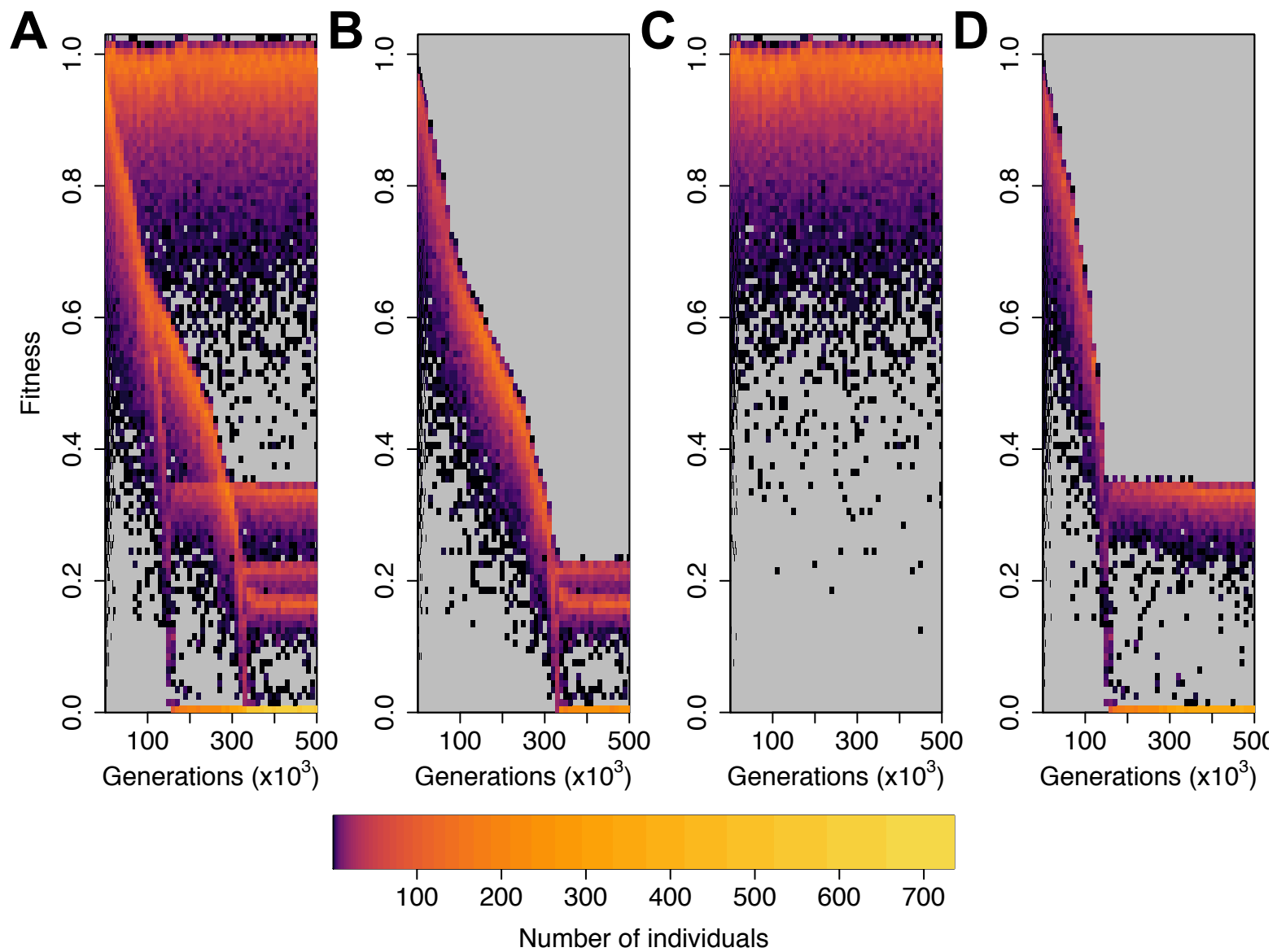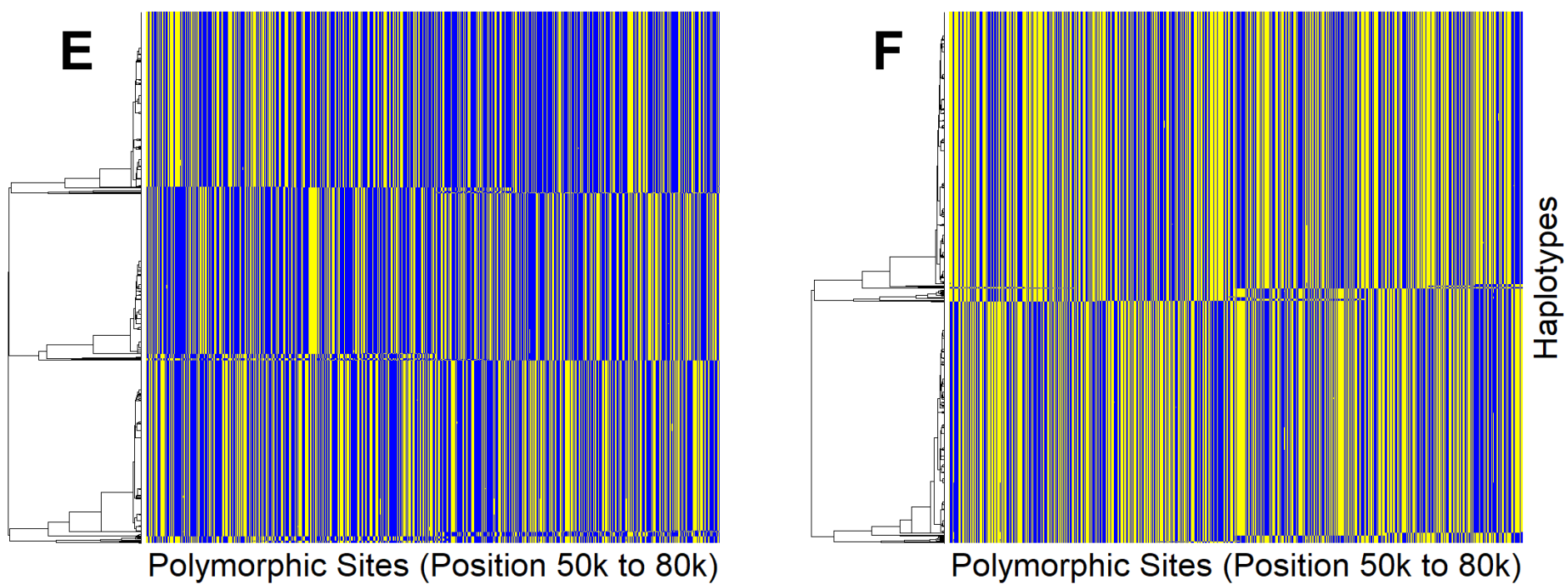

Supplement: S12 Fig — Panels A to D display the fitness distributions of A) the whole population, B) the homokaryotype for the inverted arrangement, C) the heterokaryotype and D) the homokaryotype for the standard arrangement. Panels A to D are similar to S8 Fig but for a different simulation run. Panel E) and F) correspond to the allelic content of the inverted (E) and standard arrangement (F) at generation 500,000. Each horizontal line represents a haplotype in the population and each vertical line represents a genomic position. Yellow denotes that an individual possesses the derived allele and blue the ancestral one. (PDF) [file pgen.1009411.s012.pdf]
