# Supplementary material for: Deleterious mutation accumulation and the long-term fate of chromosomal inversions
Source: PLoS Genet. 2021 Mar 4;17(3):e1009411. doi: 10.1371/journal.pgen.1009411 (PMC7963061; doi:10.1371/journal.pgen.1009411)
Supplement: S4 Fig — For A and B N = 25,000 and for C and D N = 5,000. A and C show simulations run without gene conversion and C and D show simulations with gene conversion added. All plots show distribution of the time of loss of the inversion. Simulations that remained polymorphic (cyan) or fixed (yellow) are indicated specifically to the right of the dashed line. (PDF) [file pgen.1009411.s004.pdf]

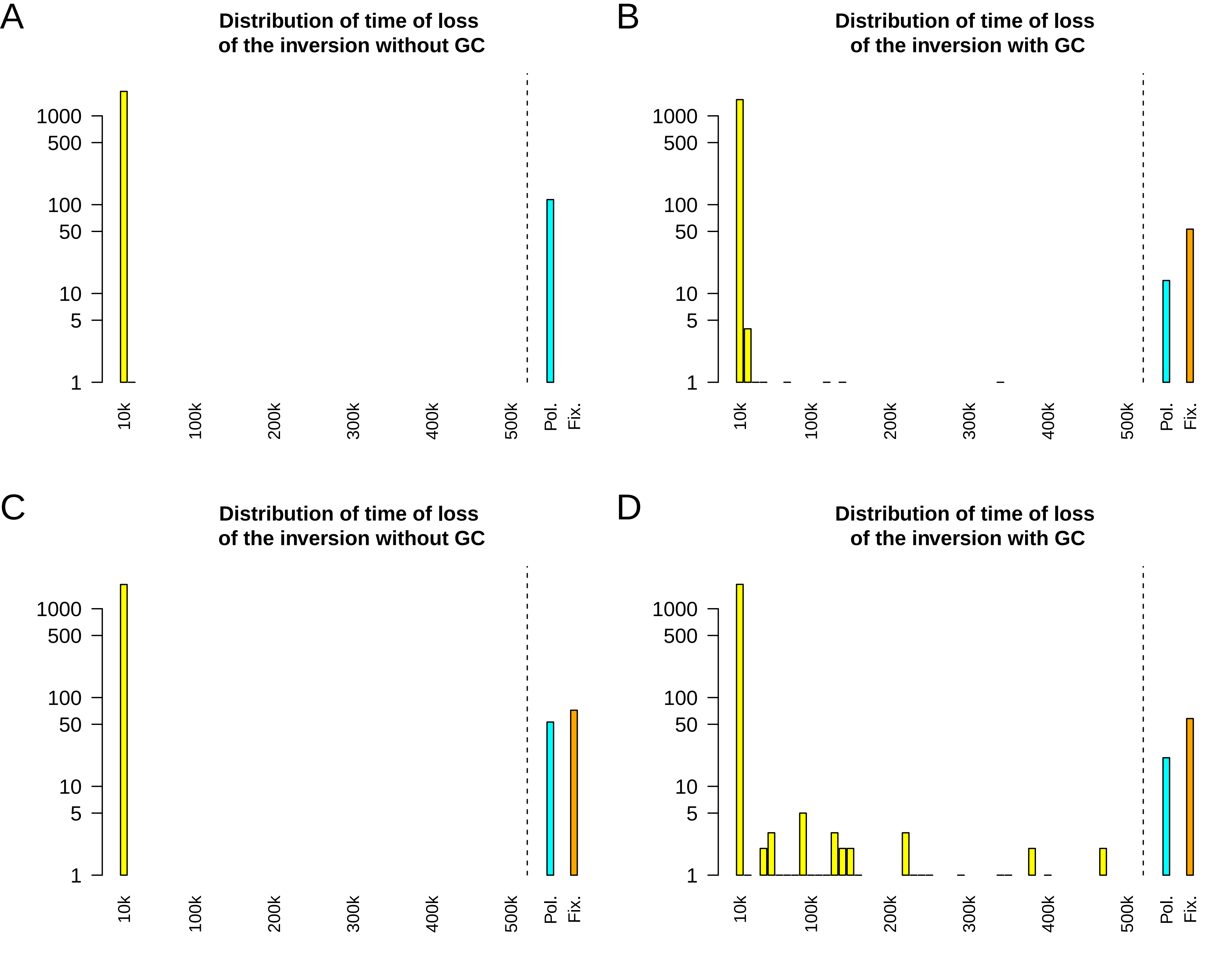
A
Distribution of time of loss of the inversion without GC
B
Distribution of time of loss of the inversion with GC
C
Distribution of time of loss of the inversion without GC
D
Distribution of time of loss of the inversion with GC
1000
500
100
50
10
5
1
10k
100k
200k
300k
400k
500k
Pol.
Fix.
